# Inhibition of human GLUT1 and GLUT5 by plant carbohydrate products; insights into transport specificity

**DOI:** 10.1038/srep12804

**Published:** 2015-08-26

**Authors:** Alayna M. George Thompson, Cristina V. Iancu, Thi Thanh Hanh Nguyen, Doman Kim, Jun-yong Choe

**Affiliations:** 1 Department of Biochemistry and Molecular Biology, Rosalind Franklin University of Medicine and Science, The Chicago Medical School, 3333 Green Bay Road, North Chicago, IL, 60064, USA; 2 The Institute of Food Industrialization, Institutes of Green Bio Science & Technology, Seoul National University, Pyeongchang-Gun, Gangwon-do, 232-916, South Korea; 3 Graduate School of International Agricultural Technology, Seoul National University, Pyeongchang-gun, Gangwon-do, 232-916, South Korea

## Abstract

Glucose transporters GLUT1 (transports glucose) and GLUT5 (transports fructose), in addition to their functions in normal metabolism, have been implicated in several diseases including cancer and diabetes. While GLUT1 has several inhibitors, none have been described for GLUT5. By transport activity assays we found two plant products, rubusoside (from *Rubus suavissimus)* and astragalin-6-glucoside (a glycosylated derivative of astragalin, from *Phytolacca americana*) that inhibited human GLUT5. These plants are utilized in traditional medicine: *R. suavissimus* for weight loss and *P. americana* for cancer treatment, but the molecular interactions of these products are unknown. Rubusoside also inhibited human GLUT1, but astragalin-6-glucoside did not. *In silico* analysis of rubusoside:protein interactions pinpointed a major difference in substrate cavity between these transporters, a residue that is a tryptophan in GLUT1 but an alanine in GLUT5. Investigation of mutant proteins supported the importance of this position in ligand specificity. GLUT1_W388A_ became susceptible to inhibition by astragalin-6-glucoside and resistant to rubusoside. GLUT5_A396W_ transported fructose and also glucose, and maintained inhibition by rubusoside and astragalin-6-glucoside. Astragalin-6-glucoside can serve as a starting point in the design of specific inhibitors for GLUT5. The application of these studies to understanding glucose transporters and their interaction with substrates and ligands is discussed.

Transport of carbohydrates across cell membranes is an important process for both normal cellular metabolism and disease states. In mammals, passive carbohydrate transport occurs through the glucose transporter (GLUT, SLC2) family^1^. In humans, there are 14 GLUT proteins, highly similar in amino acid sequence, but with various substrate specificity, tissue distribution, and regulation^2^,^3^.

GLUT1 transports glucose and is expressed in most tissues^4^,^5^. Alterations in normal glucose transport are associated with many pathologies. For example, GLUT1 is overexpressed in various cancerous tissues^6^, where it provides glucose to satisfy the extra energy requirements of cancer cells. GLUT1 overexpression may be associated with obesity and non-insulin dependent diabetes^7^, although whether this is a cause or correlation is unknown.

GLUT5 is normally expressed in the small intestine, where it absorbs fructose from the lumen^8^. Increased fructose consumption can cause deleterious metabolic effects, so GLUT5 is increasingly important for human health. Unlike glucose, fructose in serum is not regulated by insulin. At the organism level, increased fructose consumption is correlated with lipogenesis and triglyceride production, leading to insulin resistance^9^,^10^. GLUT5 is also overexpressed in some cancerous tissues, particularly breast cancer^11^.

Among GLUTs, GLUT1 is arguably the most studied and several inhibitors for its activity have been described, including forskolin and cytochalasin B^12^. Consistent with the significant sequence conservation within the GLUT family, known GLUT inhibitors often affect more than one family member. For instance, forskolin and cytochalasin B inhibit other glucose transporters, such as human GLUT2 and GLUT4^13^ and even the bacterial glucose/H^+^ symporter GlcP_Se_^14^, though not GLUT5^15^. Given its limited tissue expression and particular pattern of overexpression in diseases, GLUT5 could be an important target for therapeutic intervention, however no inhibitor of its activity has been reported. In general, finding ligands specific for a single GLUT protein would be a significant step forward in the development of therapeutic inhibitors of GLUTs. In particular, as GLUT1 is ubiquitously expressed in adult humans, viable drugs against GLUT5 should minimally impact GLUT1.

Here we report our studies on two natural products that inhibit transport by GLUT1 and GLUT5. Rubusoside (Rub) is a natural sweetener from the Chinese sweet tea plant (*Rubus suavissimus*)^16^ and closely related to glucosides found in stevia leaf (*Stevia rebaudiana*)^17^. Teas made from the leaves of *Rubus suavissimus* have been shown to be associated with caloric restriction to aid in the weight loss by obese individuals^18^. Astragalin-6-glucoside (Ast6G) is a 6-glycosylated derivative of the flavonoid astragalin^19^, a product from the American pokeweed, *Phytolacca americana*^20^. Astragalin has anti-inflammatory properties and anti-cancer therapeutic activities^21^,^22^. Glycosylated products have greater solubility; astragalin is insoluble in water, while Ast6G is water soluble and has been shown to have increased redox scavenging properties as compared to astragalin^19^.

We show that Rub inhibited transport by both GLUT1 and GLUT5, while Ast6G only inhibited GLUT5. Through *in silico* modeling of inhibitor binding we found that Rub binds in different conformations to the active sites of GLUT1 and GLUT5 due to a key residue that is a tryptophan in transporters of glucose (GLUT1-4) but an alanine in the transporter of fructose GLUT5. To explore the importance of this residue for ligand specificity, we mutated it in GLUT1 and GLUT5, by swapping tryptophan and alanine. We found that GLUT1_W388A_ still transported glucose, but became susceptible to inhibition by Ast6G and was no longer inhibited by Rub, while GLUT5_A396W_ was still inhibited by Ast6G and Rub. Interestingly, the latter mutant loosened its substrate specificity and transported not only GLUT5’s native substrate (fructose) but also glucose.

## Results

### Screening of natural products for inhibition of GLUT1 and GLUT5 transport

Human GLUT1 and GLUT5 were expressed recombinantly in insect cell culture. The purified proteins were reconstituted into proteoliposomes. To measure the inhibition of GLUT1 or GLUT5 transport activity we used entrance counter-flow transport assay^23^. In GLUT1 and GLUT5 proteoliposomes, substrate transport reached steady state after one minute incubation at room temperature.

**Figure 1:**
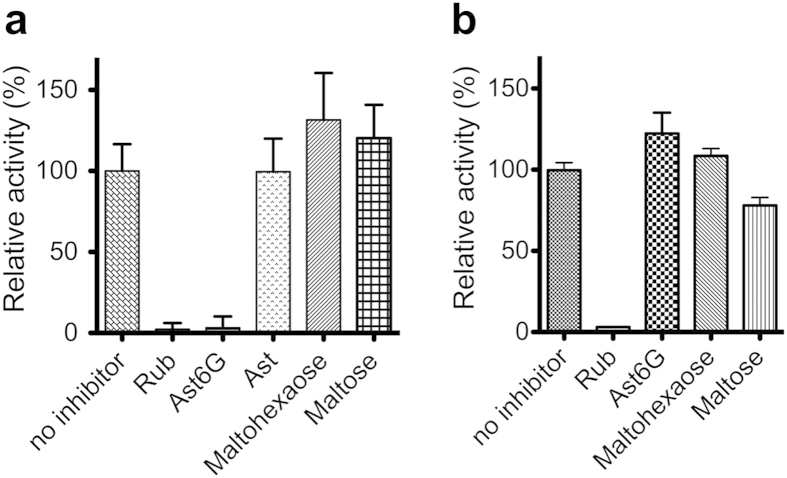
Screen for GLUT inhibition. Relative transport activity of GLUTs in proteoliposomes in the presence of inhibitors, using the entrance counter-flow transport assay. Each point is an average of at least three measurements and error bars represent standard deviation. All inhibitors were added at 20 mM. Rub, Ast and Ast6G are abbreviations for rubusoside, astragalin and astragalin-6-glucoside, respectively. (**a**) GLUT5-mediated fructose transport. (**b**) GLUT1-mediated glucose transport.

To determine GLUT5 inhibitors, we screened its relative fructose transport activity in the presence of 36 natural products (Table 1 and Fig. 1a). The majority of natural products tested did not inhibit GLUT5 activity when added at 20 mM, including astragalin, naringin, naringenin, quercetin, and stevioside. However, Ast6G and Rub did inhibit GLUT5; 20 mM Ast6G and Rub reduced GLUT5 activity to less than 5% of maximum levels (Fig. 1a). Additionally, we tested the effect of oligosaccharides on GLUT5 activity; neither maltose nor maltohexaose (6 glucose units) inhibited GLUT5 (Fig. 1a). To test if these compounds were selective for GLUT5, we measured their effect on glucose transport by GLUT1. GLUT1 was not inhibited by 20 mM maltohexaose or Ast6G, but 20 mM maltose and Rub did inhibit GLUT1 transport activity, to ~80% and 5%, respectively (Fig. 1b). Some of the conditions tested resulted in higher activity of GLUTs (maltohexaose and GLUT5; Ast6G and GLUT1, Fig. 1). It is unclear if these molecules are actually stimulating transport activity by GLUTs, nonetheless, they do not inhibit GLUT transport activity.

### Rub inhibits hexose transport by GLUT5 and GLUT1

**Figure 2:**
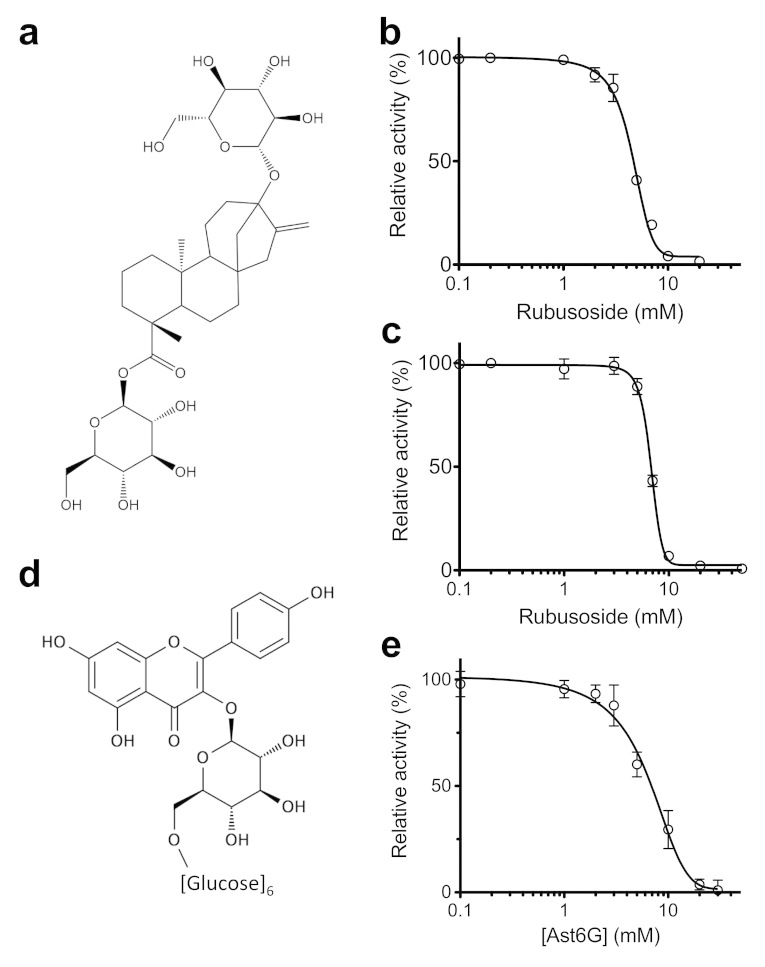
Inhibition curves of GLUT1 and GLUT5 by Rub and Ast6G. The inhibition in proteoliposomes was measured by the entrance counter-flow assay. Each data point is the average of at least three measurements and error bars represent standard deviation. Line shows non-linear fit for IC_50_ calculation. (**a**) Structure of Rub. (**b**) Inhibition of GLUT1-mediated glucose transport by Rub. (**c**) Inhibition of GLUT5-mediated fructose transport by Rub. (**d**) Structure of Ast6G; six glucose units are attached to the astragalin flavonoid head group. (**e**) Inhibition of GLUT5-mediated fructose transport by Ast6G.

Rub is a glycosylated steviol (Fig. 2a). Utilizing the entrance counter-flow assay we measured Rub-induced inhibition of glucose transport by GLUT1 (Fig. 2b) or fructose transport by GLUT5 (Fig. 2c). We found that Rub inhibits GLUT1 and GLUT5 with an IC_50_ of 4.6 ± 0.3 mM (Fig. 2b) and 6.7 ± 0.2 mM (Fig. 2c), respectively.

### Ast6G inhibits GLUT5 but not GLUT1

Ast6G is a derivative of astragalin with 6 glucosides attached in an α1→6 linkage (Fig. 2d). By entrance counter-flow transport assay, Ast6G inhibited GLUT5 mediated fructose transport with an IC_50_ of 6.8 ± 1.6 mM (Fig. 2e), but did not inhibit GLUT1 mediated glucose transport (Fig. 1b).

### Modeling of Rub interaction with GLUT1 and GLUT5

**Figure 3:**
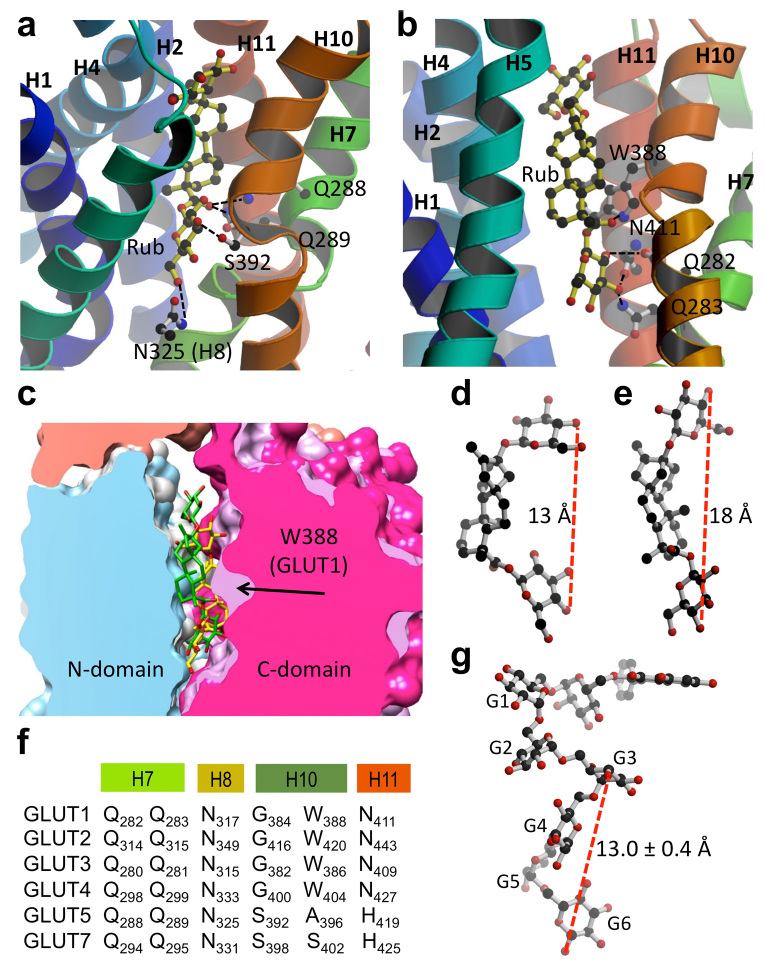
Rub binding to GLUTs. (**a**) and (**b**) Cytosolic face of GLUT is oriented towards the top, and helices 8 and 9 have been removed for better visualization. Images were generated with MolScript^32^ and raster3D^33^. (**a**) Modeled GLUT5 with docked Rub. Predicted Rub-interacting residues are: Q288 and Q289 from helix 7, N325 from helix 8, and S392 from helix 10. (**b**) GLUT1 (PDB ID 4PYP) with docked Rub. Predicted interacting residues are: Q282 and Q283 from helix 7, W388 from helix 10, and N411 from helix 11. (**c**) Comparison of space-filling models of GLUT1 (white in N-domain, light purple in C-domain) and GLUT5 (blue in N-domain, magenta in C-domain) interacting with Rub (shown as stick molecule, in yellow for GLUT1, and green for GLUT5). Molecular graphics and analyses were performed with the UCSF Chimera package^34^. (**d**) Rub as it docked to GLUT5 binding site. The distance between 4-oxygen positions of the end glucosides is 13 Å. (**e**) Rub as it docked to GLUT1 binding site. The distance between 4-oxygen positions of the two glucosides is 18 Å. (**f**) Alignment of GLUT1-5, 7 residues predicted to interact with Rub. (**g**) Model of Ast6G, with the astragalin head modeled onto isomaltooctaose chain from PDB ID 3WNN.

Using Molecular Operating Environment^24^, we simulated the interaction of Rub with GLUT1 and GLUT5 (Fig. 3). With the function ‘Dock’, Rub was docked onto the GLUT1 crystal structure (PDB ID 4PYP) or a homology model of GLUT5 (based on GLUT1 structure). GLUTs are composed of 12 transmembrane helices organized as two 6-helices bundles, the N- and C-domains, related by pseudo two-fold symmetry. In both simulations, Rub binds within the transmembrane cavity between the N- and C-domains, where it makes polar contacts with residues in the C-domain: in GLUT5 with Q288 and 289 from transmembrane (TM) helix 7, N325 from TM helix 8, and S392 from TM helix 10 (Fig. 3a); in GLUT1 with Q282 and Q283 from TM helix 7, W388 from helix 10, and N411 from helix 11 (Fig. 3b). Some of these amino acids are strictly conserved across GLUT proteins, such as Q282 and Q283 (GLUT1 numbering) which are key residues for glucose binding^14^. Others are variable, like A396 in GLUT5, which aligns with W388 in GLUT1 (Fig. 3f).

The overlaid docked interaction of Rub with GLUT1 and GLUT5 is shown in Figure 3c. In a space filling representation, the overall substrate cavity surfaces for GLUT1 and GLUT5 look similar, with the exception of GLUT1 W388; in GLUT5 this position is occupied by an alanine (Fig. 3f). The bulky tryptophan at this position near the glucose binding site could significantly influence ligand binding. Indeed, the conformations of Rub docked in GLUT1 or GLUT5 are different (Fig. 3d,e). In GLUT5, Rub is in a curved conformation, ~13 Å long (Fig. 3d). In GLUT1, Rub binds in an extended conformation, ~18 Å long (Fig. 3e). Rub does not adopt the semicircle conformation, likely because the bulky side-chain of W388 restricts the available space in the substrate cavity.

We attempted to dock Ast6G onto GLUT5 or GLUT1 structures but were unsuccessful. We were not able to generate any possible three-dimensional conformations of Ast6G using Conformation Search, and it is not possible to predict binding based on the chemical structure alone with no three-dimensional data. As Ast6G has an isomaltohexaose moiety, we looked into the structures of other oligosaccharides bound to proteins. There are several deposited protein crystal structures with isomaltooligosaccharides (PDB ID 3WNL, 3WNM, 3WNN, 3WNP and 4BFN). All of these oligosaccharides have a similar three-dimensional shape, with 4 glucosides forming a semicircle, and the average distance between 1 or 4-oxygen of glucoside G_n_ to 1-oxygen of glucoside G_n+3_ of 13.0 ± 0.4 Å, over 30 different measurements. We modeled the three-dimensional structure of Ast6G based on the isomaltooctaose structure from PDB ID 3WNN. Remarkably, the semicircle shape and approximate length of the modeled glucosidic moiety of Ast6G (Fig. 3g) were similar to those of Rub docked to GLUT5 (Fig. 3d).

### Substrate transport and inhibition of GLUT1_W388A_ and GLUT5_A396W_

The change in the substrate cavity landscape due to having a bulky tryptophan side chain in GLUT1 (W388) versus a small alanine in GLUT5 (A396) predicts different conformations for the same ligand (Rub) in these transporters. Furthermore, the tryptophan is present in transporters of glucose in the GLUT family whereas smaller side chains like alanine or serine are in the same position in GLUT5 or GLUT7, which transport fructose (Fig. 3f). Therefore, we wanted to investigate the importance of this position in substrate and inhibitor specificity.

**Figure 4:**
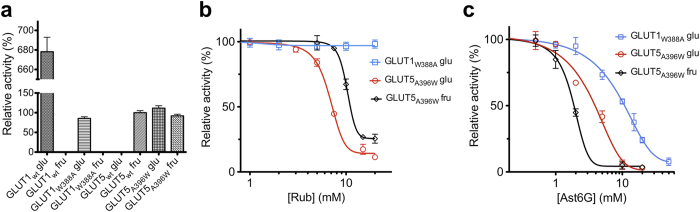
Substrate transport and inhibition of GLUT1_W388A_ and GLUT5_A396W_. Hexose transport activity of wild-type (GLUT_wt_) and mutant GLUTs was measured by entrance counter-flow transport assay. Each point is the average of three measurements and error bars represent standard deviations. Line shows non-linear fit for IC_50_ calculation. “glu” represents glucose transport; “fru” represents fructose transport. (**a**) Comparison of glucose and fructose transport of GLUT1_wt_, GLUT1_W388A_, GLUT5_wt_, GLUT5_A396W_. All measurements were normalized to GLUT5_wt_ fructose transport as 100%. Blank spaces represent assays in which no transport activity was measureable. (**b**) Concentration-dependent Rub inhibition of the relative transport activity mediated by GLUT1_W388A_ and GLUT5_A396W_. (**c**) Concentration-dependent Ast6G inhibition of the relative transport activity mediated by GLUT1_W388A_ and GLUT5_A396W_.

We expressed and purified GLUT1_W388A_ and GLUT5_A396W_. Purified mutant proteins were reconstituted into proteoliposomes and activity was determined by entrance counter-flow transport assay. Comparing wild-type GLUT1 and GLUT5 shows that GLUT1 transports ~6-fold more glucose than GLUT5 transports fructose after one minute (Fig. 4a). GLUT1 had no measurable fructose transport, nor did GLUT5 transport glucose. GLUT1_W388A_ retained glucose transport with reduced activity, ~12% transport activity of wild-type (Fig. 4a), and had no detectable fructose transport. GLUT5_A396W_ transported both glucose and fructose (Fig. 4a), with similar rates to those of the wild-type GLUT5 fructose transport and GLUT1_W388A_ glucose transport.

Next we examined the inhibition of hexose transport by GLUT1_W388A_ and GLUT5_A396W_ (Fig. 4b,c). Glucose transport by GLUT1_W388A_ was not inhibited by any tested Rub concentrations, up to 20 mM. However, both glucose and fructose transport by GLUT5_A396W_ were inhibited by Rub, with IC_50_ of 6.8 ± 0.4 mM (glucose) and 10.3 ± 0.5 mM (fructose). Unlike wild-type, GLUT1_W388A_ was inhibited by Ast6G with an IC_50_ of 10.7 ± 2.5 mM. For GLUT5_A396W_, both the glucose and fructose transport were inhibited by Ast6G, with IC_50_ of 3.7 ± 1.2 mM (glucose) and 1.8 ± 0.3 mM (fructose).

## Discussion

In humans, GLUT1 is ubiquitously expressed, while GLUT5 is normally expressed in the jejunum. GLUT5 is upregulated in several disease states, including diabetes and some breast cancers, so it is an attractive target for therapeutic intervention. Finding an inhibitor that specifically inhibits GLUT5 without affecting other GLUTs could be challenging, given the significant sequence identity among members of the family.

We report two new GLUT family inhibitors derived from natural products which inhibit hexose transport with mM IC_50_, but crucially one of them, Ast6G, inhibits only GLUT5, not GLUT1. This specificity seems at odds with the low affinity interactions implied by the IC_50_ values, however, GLUT1 and GLUT5 specifically transport their substrates with high K_m_ (GLUT1 K_m_ for glucose is ~3 mM^25^, while GLUT5 K_m_ for fructose is ~10 mM^15^). Ast6G and Rub contain glucoside moieties, so the interaction of inhibitor and protein likely mirrors the energetics of substrate and protein interactions.

Ast6G inhibits GLUT5, but not GLUT1 (Figs 1 and 2). The inhibition of GLUT5 activity by Ast6G is efficient; activity is reduced to ~5% of maximum, and the IC_50_ (~6 mM) is similar to reported K_m_ for fructose (~10 mM)^15^. As neither maltohexaose nor astragalin alone inhibit GLUT5 (Fig. 1a), it seems that concurrent interactions with the two moieties of Ast6G are needed for transport inhibition of GLUT5. Rub inhibits both GLUT1 and GLUT5 (Fig. 2). It is an efficient inhibitor, reducing activity to background levels, and its IC_50_ (~5 mM for both proteins) is also similar to reported K_m_ values for glucose transport by GLUT1^25^.

Based on *in silico* docking, we predict that Rub binds to GLUT1 and GLUT5 near the transmembrane substrate binding site, but that Rub is in different conformations and makes different molecular contacts with GLUT1 as compared to GLUT5 (Fig. 3). A crucial difference between GLUT1 and GLUT5 transmembrane cavities is W388 in GLUT1 versus A396 in GLUT5, which is near the predicted Rub binding site in both proteins (Fig. 3c). Even with different amino acid contacts, it seems that Rub could interfere with substrate transport by two different GLUTs because it blocks access to the transmembrane substrate cavity by interacting with two key conserved glutamines from TM helix 7 (Fig. 3).

Interestingly, W388 of GLUT1 is conserved in GLUT1-4 which primarily transport glucose, whereas A396 of GLUT5 aligns with S402 in GLUT7, a GLUT that transports both fructose and glucose^26^. Therefore, this position was investigated for its importance in ligand specificity. We mutated W388 of GLUT1 to alanine and A396 of GLUT5 to tryptophan and checked for changes in the transport or inhibitor specificity. GLUT1_W388A_ retained glucose transport activity, though significantly decreased as compared to wild-type, while the complementary mutant of GLUT5_A396W_ retained fructose transport but gained glucose transport activity (Fig. 4a). Rub inhibited glucose- and fructose-transport by GLUT5_A396W_, with an increased IC_50_ compared to wild-type (10 mM vs. 5 mM for fructose transport), but did not inhibit glucose transport by GLUT1_W388A_ (Fig. 4b). This result suggests that W388 of GLUT1 is critical for inhibition by Rub, consistent with the docking of Rub to GLUT1, which predicts that W388 directly interacts with Rub (Fig. 3b). In GLUT5, the interpretation is more complicated; we could conclude that A396 is not involved in Rub interaction, but is more likely that replacing A396 with tryptophan does not disrupt Rub normal binding mode in GLUT5.

Unlike wild-type GLUT1, GLUT1_W388A_ is susceptible to inhibition by Ast6G. Also, both glucose- and fructose-transport activities of GLUT5_A396W_ are inhibited by Ast6G with lower IC_50_ than wild-type GLUT5 (Figs 2e and 4c). Taken together, these data suggest that W388/A396 is involved in but not solely responsible for the Ast6G discrimination between GLUT1 and GLUT5. Based on the structures of isomaltohexaoses bound to proteins, we modeled a possible conformation of Ast6G; this structure is ~25 Å long (Fig. 3g). Ast6G likely interacts with several different areas of GLUT5, including the proximity of A396, which allows recognition and inhibition of GLUT1_W388A_.

Importantly, a single A→W mutation has changed the substrate specificity of a GLUT protein, opening doors to understanding the specificity of substrate interactions in this complex and essential family group. Why the mutation changed substrate specificity in GLUT5 but not GLUT1 is unclear, but it can be inferred that W388 is not an essential residue in transport, but is rather involved in substrate recognition. This idea is supported by modeling studies by Madej *et al.*, who speculated that tryptophan or alanine could provide a molecular switch between GLUT1 and GLUT5 substrate recognition^27^. They docked glucose into GLUT1 and fructose into GLUT5 and found that substitution of alanine for the bulky tryptophan allows space for the 6-OH of fructose in the binding site of GLUT5. Crucially missing from both the simulations and our current results is an understanding of substrate recognition by GLUTs that transport both glucose and fructose (GLUT2, GLUT5_A396W_, and GLUT7). GLUT2 and GLUT5_A396W_ have W388 equivalents, while GLUT7 contains a serine. Based on Madej’s work, it seems that the transmembrane binding site allows some flexibility for sugar binding, as glucose in GLUT1 and fructose in GLUT5 are found in slightly different orientations. Previous work has identified I314 from GLUT7 as crucial to fructose, but not glucose, transport^28^. Perhaps the answer to transport of multiple substrates is different binding modes and/or sites. Further studies are needed to elucidate substrate recognition in this important family of transporters.

It seems likely that the beneficial effects of Rub and Ast6G are due at least in part to their inhibition of GLUT1 and GLUT5. Rub is not selective for either GLUT1 or GLUT5, though it probably interacts differently with each protein at a molecular level. Ast6G specifically inhibits GLUT5, and we propose that sequence divergence around the substrate-binding site explains this specificity. To develop a specific GLUT5 inhibitor with clinical implications requires more studies, but the lessons learned here should inform further work.

## Methods

### Protein Expression and Purification

cDNAs of GLUT1 and GLUT5 were purchased from Open Biosystems (GE Healthcare). Full length DNA was subcloned into pFastBac1 (Life Technologies) vector with a N-terminal hexahistidine tag. Bacmids were generated in DH10Bac *E. coli* cells (Life Technologies). Baculoviruses were produced using Cellfectin II Reagent and amplified in Sf21 insect cells (Life Technologies). Cells were maintained at 26 °C, and P1 (10^6^ pfu/mL) was collected from infected cells after 72 hours. Sf21 cells were propagated in HyClone SFX-Insect media (GE Healthcare), supplemented with 5% fetal bovine serum (Biowest), antibiotics (100 units/mL Penicillin G and 100 μg/mL streptomycin sulfate) and amphotericin B (2.5 μg/mL). For recombinant protein expression, Sf21 cells in suspension culture at 2 × 10^6^ cells/ml were infected with P3 viral stock (10^8^ pfu/mL), at an MOI of 1.0 pfu/cell. Four days after viral infection, cells from 2 L culture were collected by centrifugation at 2,000 × g and 25 °C. The cell pellet was resuspended in buffer A containing 50 mM sodium phosphate (NaP_i_) (pH 7.5) 5% (v/v) glycerol, 200 mM NaCl and protease inhibitors (1 mM AEBSF, 10 μM E-64, 10 μM pepstatin A, 1 μM Aprotinin, 20 μM Bestatin, 20 μM Leupeptin) at 4 °C, and disrupted by sonication (Branson Ultrasonic, Danbury, CT). Cell debris was removed by centrifugation at 12,000 × g and 4 °C for 30 minutes. The membrane fraction was collected by ultracentrifugation at 200,000 × g and 4 °C for 3 hours, and solubilized with 1% (w/v) n-dodecyl-β-D-maltopyranoside (DDM, EMD chemicals) in 120 mL of buffer A at 4 °C for 2 hours. The solubilized membranes were subject to ultracentrifugation at 200,000 × g for 30 minutes, and the supernatant was loaded onto the Talon metal affinity resin (Clontech) and washed with buffer containing 50 mM NaP_i_ (pH 7.5) 500 mM NaCl, 5–10 mM imidazole, 5% (v/v) glycerol and 0.05% (w/v) DDM. GLUT1 (or GLUT5) was eluted with 50 mM NaP_i_ (pH 7.5) 5% (v/v) glycerol, 200 mM NaCl, 200 mM imidazole and 0.05% DDM (w/v). Eluted protein was incubated overnight at 4 °C with thrombin (BioPharm Laboratories) to remove the N-terminal poly-His tag. The thrombin-transporter digestion was loaded again on Talon affinity resin, after lowering the imidazole concentration to 2 mM in the loading buffer. Pure protein was collected from the flow-through. To generate DNA for mutant proteins, site-directed mutagenesis was performed on the pFastBac1 plasmid constructs of wild-type proteins and verified by DNA sequencing^29^. Mutant proteins were purified in the same manner as wild-type with no modifications.

### Preparation of proteoliposomes and counter-flow hexose transport assay

Proteoliposomes were generated according to the protocol from^30^ with minor modifications. Liposomes were produced from a 95/5% (w/w) mix of soy phosphatidylcholine and cholesterol (Avanti Polar Lipids). Briefly, hexane-washed and vacuum-dried lipids were resuspended to 20 mg/ml in 100 mM KP_i_ (pH 7.5), and then subjected to 11 freeze-and-thaw cycles in liquid nitrogen and room temperature water. The lipid mixture was extruded through a 0.1 μm polycarbonate filter. Prepared liposomes were destabilized with 4 mM Triton X-100 and mixed with purified protein in a 100:1 (w/w) ratio in 100 mM KP_i_ (pH 7.5) 20% (v/v) glycerol, 200 mM (glucose or fructose). Detergent was removed by several additions of SM2 BioBeads (BioRad) and incubated overnight at 4 °C. After filtering out the BioBeads, proteoliposomes were diluted with 100 mM KP_i_ (pH 7.5) 200 mM (glucose or fructose), and then collected by ultracentrifugation, at 200,000 × g and 4 °C, for 1 hour. The proteoliposomes pellet was resuspended in the above buffer so that O.D._600nm_ was ~30.

To measure hexose transport, we utilized entrance counter-flow assay^23^. Transport was performed in a volume of 100 μL of 100 mM KP_i_ (pH 7.5) buffer containing radioactive ^14^C-hexose (4 μM fructose or 5 μM glucose) with inhibitors added before initiation of transport. Radioactive substrate uptake in GLUT1 and GLUT5 (wild-type or mutant) proteoliposomes was constant between 1 and 2 mins. The reaction was started by the addition of 3 μL concentrated proteoliposomes to the above solution and incubated at room temperature; the transport was stopped after one minute by addition of ice-chilled quench buffer [100 mM KP_i_ (pH 5.5) and 100 mM LiCl]. The solution was filtered through a 0.4 μm pore size cellulose nitrate membrane filter (Whatman), and the filter was washed three times with quench buffer. The membrane filter was placed into a vial filled with BioSafe II scintillation liquid (Research Products International Corp.), and radioactivity was quantified with LS 6500 scintillation counter (Beckman). Some of the natural products tested were not water soluble at high concentrations, but were soluble in DMSO (see Table 1). The transport assays were unaffected by 5% DMSO. Data is presented as relative activity normalized to radioactivity of no inhibitor added as 100% and empty proteoliposomes as 0%. Kinetic parameters were fitted by nonlinear algorithm plots using Prism (GraphPad Software).

### Preparation of rubusoside and astragalin-6-glucoside

Rubusoside (Rub) was prepared from the stevioside mixture and lactase from *Thermus thermophilus* as reported previously^17^. Briefly, the reaction mixture containing 20 mL of 1% (w/v) stevioside (Ste) solution in 40 mM Tris-HCl buffer (pH 7.0) and 10 mL alginate beads (300 U lactase/mL beads) were incubated at 70 °C in a water bath. After one day of enzymatic reaction, produced Rub was checked by TLC. For the purification of Rub, 20 mL (100 g/mL) of Ste reaction digest was applied to Reveleris® Amino 80 g Flash Cartridges (Grace Discovery Science, Shanghai, China) and Rub was detected with an evaporative light scattering detector (ELSD). A mixture of acetonitrile and water was used as an eluent with a gradient from 95: 5 (v/v) to 50:50 (v/v) of acetonitrile: water at a flow rate of 60 mL/min and room temperature. Purified Rub eluted at 42 minutes; the fractions were pooled and freeze-dried for further study.

Kaempferol and astragalin-6-glucoside were prepared as reported previously^19^. The reaction mixture in 20 mM Na-Ac buffer (pH 5.2) and 10% (v/v) DMSO containing 10 mM astragalin, 200 mM sucrose, and 512FMCM dextansucrase (1.8 U/mL) from *Leuconostoc mesenteroides* was incubated at 28 °C for 5 hours. Acceptor product was purified by chromatography on a Sephadex LH-20 gel column with a gradient of 0–100% ethanol.

### Modeling of Rub interaction with GLUT1 and GLUT5

GLUT5 homology model was built in Coot^31^, based on the GLUT1 structure (PDB ID 4PYP); the amino acid substitution was guided by sequence alignment [amino acid sequence identity between GLUT1 and GLUT5 is 40% (the similarity is 63%) as calculated by Vector NTI (Life Technologies)]. When only the transmembrane helices were considered, sequence identity between GLUT1 and GLUT5 increased to 50%. GLUT5 model thus obtained was subject to energy minimization with Molecular Operating Environment (MOE)^24^.

Interaction of Rub with GLUT5 model and GLUT1 structure (PDB ID 4PYP) was modeled using MOE^24^. A library of 21 possible conformations of Rub was generated with Conformation Search using the LowMode MD algorithm (no rigid-body, no fixed O-H bond lengths, unconstrained double-bond rotation). Possible ligand binding sites were determined with SiteFinder; for each transporter, several possible sites were identified, but the internal site near the transmembrane binding site (including conserved glutamines Q282 and 283 in GLUT1; Q288 and 289 in GLUT5) was chosen for docking; dummy atoms were placed at this site for docking. Before docking, proteins were prepared by protonation at pH 7.5 and then energy minimization. Rubusoside conformations were docked onto the energy-minimized protein structures with Dock with all default parameters in Triangle Matcher retaining 100 poses with London dG scoring (estimates free energy of binding, based upon entropy changes, loss of flexibility of the ligand, hydrogen bond geometry, and desolvation of all atoms) and refined, retaining 30 poses using Alpha HB rescoring (with equal weights for hydrogen bonds and geometry of ligand-receptor fit). After docking, poses were sorted by ascending refinement score, and top 15 (or fewer, if scores became positive) scored poses were screened for reasonable interactions with the protein based on physiochemical properties.

Attempts to dock Ast6G were unsuccessful because three-dimensional conformations of the ligand were not generated using Conformation Search. We searched using all algorithms available; LowMode MD, Stochastic, and Systematic, with more than 100,000 iterations in each search.

## Additional Information

**How to cite this article**: George Thompson, A. M. *et al.* Inhibition of human GLUT1 and GLUT5 by plant carbohydrate products; insights into transport specificity. *Sci. Rep.* **5**, 12804; doi: 10.1038/srep12804 (2015).

## Figures and Tables

**Table 1:** List of compounds screened for GLUT5 fructose transport inhibition.

Epigallocatechin gallate (10 mM)	Arbutin (20 mM)
Astragalin (D^1^, 20 mM)	Astragalin 1 glucoside (20 mM)
Rutin (D^1^, 20 mM)	Astragalin 2 glucoside (20 mM)
Gallic acid (D^1^, 20 mM)	**Astragalin 6 glucoside** (20 mM)
Naringin (5 mM)	Fructose oligosaccharides (20 mM)
Naringenin (D^1^, 20 mM)	Megafructose oligosaccharides (10 mM)
Catechin hydrate (D^1^, 20 mM)	Bitter mellon (10 mg/ml)
Curcumin (D^1^, 20 mM)	Rice wine ethanol extraction (10 mg/ml)
Korean herbal wine (Bekseju) ethanol extraction (10 mg/ml)	Korean herbal wine (Bekseju) water extraction (10 mg/ml)
Dihydromyricetin (D^1^, 20 mM)	Stevioside (20 mM)
Ampelopsin glucoside 1 (10 mM)	Cycloisooligosaccharide (10 mM)
Epigallocatechin gallate glucoside 1 (10 mM)	Human milk oligosaccharide mixture (without lactose) (10 mg/ml)
Quercetin (D^1^, 20 mM)	Maltose (20 mM)
Pyrogallol (20 mM)	Maltotriose (20 mM)
Pyrocatechol (20 mM)	Maltotetraose (20 mM)
Chrysin (5 mM)	Maltopentaose (20 mM)
Hydroquinone (5 mM)	Maltohexaose (20 mM)
Ascorbic acid (20 mM)	**Rubusoside** (20 mM)

In parenthesis is the maximum concentration used for each compound. D^1^ indicates that the compound was dissolved in DMSO, at a final stock concentration of 500 mM, then diluted at the indicated concentration in the assay solution. Only astragalin-6-glucoside and rubusoside inhibited GLUT5 (in bold).
